# Construction and verification of prediction model for Alzheimer’s disease in diabetic patients

**DOI:** 10.3389/fendo.2025.1699771

**Published:** 2025-11-18

**Authors:** Li Wang, Qing Sun

**Affiliations:** 1 National Heart and Lung Institute, Imperial College London, London, United Kingdom; 2 College of Business and Management, Saint Paul University Manila, Manila, Philippines

**Keywords:** diabetes, Alzheimer’s disease, nomogram prediction model, glucose and lipid metabolism, multivariate logistic regression analysis

## Abstract

**Objective:**

This study aimed to construct a prediction model for Alzheimer’s disease (AD) in diabetic patients and evaluate its clinical application value.

**Methods:**

A total of 322 patients was included and randomly divided into a training set (n=225) and a validation set (n=97) at a ratio of 7:3. Clinical characteristic data of the patients were collected. In the training set, univariate analysis and multivariate logistic regression analysis were used to identify the relevant risk factors for AD onset, and a nomogram prediction model was constructed accordingly. The receiver operating characteristic (ROC) curve and calibration curve were plotted and validated in an independent validation dataset. In addition, decision curve analysis (DCA) was used to further evaluate the application value and significance of the nomogram model in clinical practice.

**Results:**

The incidence of AD in the training set was 18.67% (42/225), and that in the validation set was 18.56% (18/97). Multivariate regression analysis showed that age, duration of diabetes, fasting plasma glucose (FPG), glycosylated hemoglobin (HbA1c), triglyceride (TG), and homeostasis model assessment of insulin resistance (HOMA-IR) were all independent risk factors for AD onset (all *P* < 0.05). In the training set and validation set, the nomogram prediction model showed good predictive performance, with the concordance index (C-index) reaching 0.868 and 0.710 respectively. Calibration curve analysis showed a high degree of agreement between the predicted values and the observed values. The mean absolute errors in the training set and validation set were 0.103 and 0.116 respectively. The results of the Hosmer-Lemeshow test were *χ²* = 10.515, *P* = 0.230 and *χ²* = 5.987, *P* = 0.648 respectively. The ROC curve showed that the AUCs of the nomogram model for predicting occurrence of AD in the training set and validation set were 0.866 (95% CI: 0.794- 0.939) and 0.718 (95% CI: 0.517-0.920) respectively.

**Conclusion:**

The prediction model for AD in diabetic patients can assist in the early prediction of the risk of AD onset, laying a solid foundation for formulating effective clinical intervention strategies. This is crucial for delaying the progression of AD and significantly improving the quality of life of patients.

## Introduction

Alzheimer’s disease (AD) is a chronic and progressive neurodegenerative disorder. Its typical symptoms involve a comprehensive decline in cognitive function, including memory loss, impaired language function, object recognition disorders, and loss of executive function, which severely impairs the patients’ quality of life (1). In recent years, the global population aging phenomenon has become more obvious. This trend has directly led to a significant annual increase in the incidence of AD, making it an urgent global public health challenge. Diabetes is characterized by a state of hyperglycemia and is highly associated with the occurrence of various secondary complications. Numerous studies have revealed a close association between diabetes and AD. Specifically, the risk of AD in diabetic patients is significantly higher than that in non-diabetic patients (2). The disorder of glucose and lipid metabolism caused by diabetes may promote the occurrence and development of AD through multiple mechanisms such as oxidative stress, inflammatory response, and insulin resistance (3). Therefore, constructing a prediction model for AD in diabetic patients is of great significance for early identification of high-risk populations for AD, formulating effective preventive measures, and delaying the progression of AD (4). Nomograms can visually display the probability of an individual’s disease risk or prognosis by integrating multiple clinical factors (5). Nomograms have been widely used in the medical field, such as in tumor prognosis prediction and cardiovascular disease risk assessment. The core purpose of this study is to construct a nomogram prediction model for predicting the risk of AD based on the clinical characteristic data of diabetic patients and indicators related to glucose and lipid metabolism, and further comprehensively evaluate the clinical practical value of this model, aiming to provide solid scientific theoretical support and practical guidance for the early identification and effective intervention of AD.

## Methods

### Study subjects

In this study, 322 diabetic patients who received diagnosis and treatment services in the endocrine specialty outpatient department of our hospital from January 2020 to December 2023 were selected. The inclusion criteria were as follows: 1) meeting the diabetes diagnosis criteria established by the World Health Organization (WHO) in 2023 (6); 2) age ≥ 40 years; 3) signing the informed consent form and voluntarily participating in this study. The exclusion criteria were as follows: 1) a history of mental illness or cognitive impairment; 2) comorbid severe diseases of organs such as the heart, liver, and kidneys; 3) suffering from other types of neurodegenerative diseases; 4) refusing to accept relevant examinations and follow-up. The participants were then randomly allocated to a training set (n=225) and a validation set (n=97) at a 7:3 ratio. This study was approved by the Ethics Committee of Hospital. Written informed consent was obtained from all participants.

### Data collection

The following clinical characteristic data were collected by reviewing the patients’ medical records and conducting questionnaires: 1) basic information such as age, gender, height, weight, smoking history, drinking history, blood pressure (systolic blood pressure (SBP), diastolic blood pressure (DBP)); 2) duration of diabetes, type of diabetes, and complications of diabetes; 3) biochemical indicators such as fasting blood glucose (FPG), glycosylated hemoglobin (HbA1c), 2-hour postprandial blood glucose (2hPG), total cholesterol (TC), triglyceride (TG), high-density lipoprotein cholesterol (HDL-C), low-density lipoprotein cholesterol (LDL-C); 4) glucose metabolism-related indicators such as the insulin resistance index (HOMA-IR).

### Criteria for occurrence of AD

All patients included in the study were evaluated for their cognitive function status using the standardized Mini-Mental State Examination (MMSE). The MMSE score ranges from 0 to 30 points, with lower scores indicating more severe cognitive impairment. A MMSE score ≤ 24 points was used as the pragmatic diagnostic criterion for AD in this study. It is important to note that while the MMSE is a widely used and practical screening tool, a diagnosis based solely on it may lack the specificity achieved by incorporating neuroimaging or cerebrospinal fluid biomarkers, which was not feasible in our clinical setting. Furthermore, our binary classification (AD vs. non-AD) did not capture the prodromal stage of Mild Cognitive Impairment (MCI), which is a critical target for early prediction. Patients with a MMSE score ≤ 24 points were diagnosed with AD, and those with a MMSE score > 24 points were diagnosed as non-AD. According to the MMSE cognitive function assessment results, the patients were divided into the AD group (42 cases) and the non-AD group (183 cases).

### Sample size estimation

Sample size calculation was performed first in this study: Based on the expected incidence of the primary outcome (Alzheimer’s disease, AD) – set to 18%-19% (per prior literature, pre-experiments, and target population characteristics, consistent with actual incidence) – a scheme matching the core statistical method (multivariate logistic regression) was adopted. Calculations were done via the “Sample Size Calculation” module in SPSS 26.0 and verified using the “pwr” package in R 4.2.1; significance level α=0.05 (two-tailed), power 1-β=80%, and 10% potential loss-to-follow-up were considered, yielding a minimum required sample size of 295 cases. Accordingly, 322 diabetic patients from the endocrine outpatient department of our hospital (January 2020–December 2023) were enrolled. The actual sample size exceeded the minimum requirement, and its statistical power (1-β>80%) was verified by SPSS 26.0, meeting the statistical needs of multivariate analysis.

### Statistical analysis

Data analysis was performed using SPSS 26.0 software and R software (version 4.3.1). Measurement data conforming to normal distribution were expressed as mean ± standard deviation, with independent sample t-test for intergroup comparison; data not conforming to normal distribution were presented as median (interquartile range) [M (Q1, Q3)], and Mann-Whitney U test was used for intergroup comparison. Enumeration data were expressed as number of cases (percentage) [n (%)], and intergroup comparison was conducted using *χ²* test or Fisher exact probability method. Unadjusted Odds Ratios (ORs) with 95% confidence intervals (CIs) were calculated for all variables during the univariate analysis. Factors with *P* < 0.05 in univariate analysis were included in multivariate logistic regression analysis to identify independent prognostic factors, and ORs and 95% CIs were calculated. Sample size calculation was conducted in accordance with prediction model guidelines, with significance level α=0.05 and power 1-β=80%. The classic principle of “at least 10 events per variable (EPV)”, 12 predictors in this study corresponded to 60 events, and model stability was verified with low inter-variable correlation (VIF<2). Nomogram models were constructed based on independent prognostic factors (implemented using the “rms” package in R software) and internally validated via Bootstrap method (1000 repeated samplings); consistency index (C-index) was calculated to assess discriminative ability, calibration curves were plotted to evaluate calibration, and receiver operating characteristic (ROC) curves with area under the curve (AUC) and 95% CI were used to assess predictive accuracy. Decision curve analysis (DCA) was used to evaluate the clinical application value of the nomogram by calculating the net benefit at different threshold probabilities. The significance level was set at α=0.05.

## Results

### Comparison of baseline characteristics between the training set and the validation set

A total of 322 diabetic patients were included and randomly divided into a training set (n = 225) and a validation set (n = 97). There were no significant differences between the two sets of patients in terms of age, gender, duration of diabetes, BMI, SBP, DBP, HbA1c, 2hPG, FPG, TC, TG, HDL-C, LDL-C, INS, and HOMA-IR, indicating comparability (all *P* > 0.05) (**Table 1**).

**Table 1:** Comparison of baseline characteristics between the training set and the validation set.

Variables		Training set (n = 225)	Validation set (n = 97)	t/χ²	*P*
Age (years)		63.50 ± 8.26	62.11 ± 8.12	0.611	0.541
BMI (kg/m^2^)		23.32 ± 2.15	23.64 ± 2.22	1.213	0.225
Duration of diabetes (years)		8.37 ± 4.24	8.23 ± 4.07	0.275	0.783
Gender	Male	120(53.33)	53(54.64)	0.046	0.829
	Female	105(46.67)	44(45.36)		
Smoking history	Yes	47(20.89)	18(18.56)	0.228	0.632
	No	178(79.11)	79(81.44)		
Drinking history	Yes	30(13.33)	12(12.37)	0.055	0.814
	No	195(86.67)	85(87.63)		
Type of diabetes	Type 1	35(15.56)	22(22.68)	2.361	0.124
	Type 2	190(84.44)	75(77.32)		
Diabetes complications	Yes	94(41.78)	38(39.18)	0.189	0.663
	No	131(58.22)	59(60.82)		
SBP (mmHg)		131.24 ± 15.37	130.75 ± 14.89	0.264	0.791
DBP (mmHg)		78.67 ± 8.23	79.02 ± 7.98	0.353	0.724
FPG (mmol/L)		7.84 ± 2.31	7.92 ± 2.25	0.287	0.774
HbA1c (%)		7.63 ± 1.25	7.62 ± 1.18	0.067	0.946
2hPG (mmol/L)		10.33 ± 3.05	10.38 ± 2.97	0.136	0.891
TC (mmol/L)		3.23 ± 1.05	3.20 ± 0.98	0.239	0.810
TG (mmol/L)		1.84 ± 0.88	1.91 ± 0.85	0.661	0.508
HDL-C (mmol/L)		1.16 ± 0.32	1.14 ± 0.34	0.504	0.614
LDL-C (mmol/L)		3.11 ± 0.81	3.08 ± 0.79	0.307	0.758
FIN (mU/L)		10.57 ± 3.52	10.83 ± 3.46	0.601	0.547
HOMA-IR		3.24 ± 1.03	3.34 ± 1.02	0.801	0.423

### Univariate analysis of risk factors for occurrence of AD in the training set

The incidence of AD in the training set was 18.67% (42/225), and that in the validation set was 18.56%. In the training set, univariate analysis showed that factors such as age, duration of diabetes, FPG, HbA1c, TG, and HOMA-IR were significantly different between occurrence of AD group and non-occurrence of AD group (all *P* < 0.05) (**Table 2**).

**Table 2:** Univariate analysis of risk factors for occurrence of AD in the training set.

Variables	Occurrence of AD group (n = 42)	Non-occurrence of AD group (n = 183)	t/χ²	*P*	Unadjusted OR (95% CI)
Age (years)	65.27 ± 7.61	61.34 ± 6.57	3.391	0.008	1.09 (1.03-1.16)
BMI (kg/m²)	22.84 ± 3.83	23.03 ± 3.64	1.288	0.199	1.06 (0.97-1.16)
Duration of diabetes (years)	12.53 ± 5.47	9.77 ± 4.12	3.666	0.001	1.15 (1.06-1.24)
Gender			0.020	0.887	
Male	23 (54.76%)	98 (53.55%)			1.05 (0.54-2.04)
Female	19 (45.24%)	85 (46.45%)			1 (Reference)
Smoking history			0.468	0.493	
Yes	10 (23.81%)	35 (19.13%)			1.32 (0.60-2.92)
No	32 (76.19%)	148 (80.87%)			1 (Reference)
Drinking history			0.356	0.550	
Yes	8 (19.05%)	28 (15.30%)			1.30 (0.55-3.08)
No	34 (80.95%)	155 (84.70%)			1 (Reference)
Type of diabetes			0.011	0.915	
Type 1	6 (14.29%)	25 (13.66%)			1.06 (0.41-2.73)
Type 2	36 (85.71%)	158 (86.34%)			1 (Reference)
Diabetes complications			0.790	0.374	
Yes	18 (42.86%)	65 (35.52%)			1.36 (0.69-2.68)
No	24 (57.14%)	118 (64.48%)			1 (Reference)
SBP (mmHg)	132.12 ± 16.65	130.54 ± 16.53	0.557	0.577	1.01 (0.99-1.03)
DBP (mmHg)	79.87 ± 9.16	78.61 ± 8.67	0.840	0.401	1.02 (0.98-1.06)
FPG (mmol/L)	8.54 ± 2.56	7.05 ± 2.38	3.607	0.001	1.28 (1.11-1.48)
HbA1c (%)	8.31 ± 1.52	7.54 ± 1.24	3.472	0.001	1.62 (1.22-2.16)
2hPG (mmol/L)	11.24 ± 2.98	10.45 ± 3.04	1.524	0.128	1.09 (0.97-1.22)
TC (mmol/L)	3.55 ± 1.26	3.46 ± 1.08	0.471	0.637	1.07 (0.80-1.44)
TG (mmol/L)	2.37 ± 1.12	1.88 ± 0.87	3.109	0.002	1.81 (1.25-2.62)
HDL-C (mmol/L)	1.04 ± 0.33	1.11 ± 0.30	1.338	0.182	0.48 (0.17-1.37)
LDL-C (mmol/L)	3.15 ± 0.95	3.12 ± 0.88	0.196	0.844	1.04 (0.70-1.55)
FINS (mU/L)	11.24 ± 3.79	10.52 ± 3.31	1.236	0.217	1.06 (0.96-1.17)
HOMA-IR	3.86 ± 1.26	3.13 ± 1.12	3.719	0.001	1.75 (1.29-2.37)

### Multivariate logistic regression analysis of risk factors for occurrence of AD

With the occurrence of AD as the dependent variable (0=no, 1=yes), in the preliminary screening stage of univariate analysis, variables with a statistically significant (*P* < 0.05) were selected as candidate covariates. The results of the multivariate logistic regression analysis showed that older age, longer duration of diabetes, higher levels of HbA1c and TG, and a higher HOMA-IR index were independent risk factors for AD occurrence (all *P* < 0.05) (**Table 3**). In the regression model, the tolerance of each variable was > 0.1, the VIF was < 2, the condition index was < 30, and there was no situation where the variance proportion of multiple covariates under the same eigenvalue was > 50%. Therefore, there was no collinearity among the covariates.

**Table 3:** Multivariate logistic regression analysis of risk factors for occurrence of AD.

Variables	*β*	*SE*	*Wald*	*P*	*OR*	95%CI
Age	0.084	0.026	10.424	0.001	1.088	1.034-1.145
Duration of diabetes	0.137	0.040	11.715	0.001	1.147	1.060-1.240
FPG	0.261	0.077	11.633	0.001	1.299	1.118-1.509
HbA1c	0.465	0.141	10.901	0.001	1.592	1.208-2.097
TG	0.576	0.191	9.082	0.003	1.779	1.223-2.586
HOMA-IR	0.550	0.160	11.893	0.001	1.733	1.268-2.370

### Development of the nomogram prediction model

**Figure 1:**
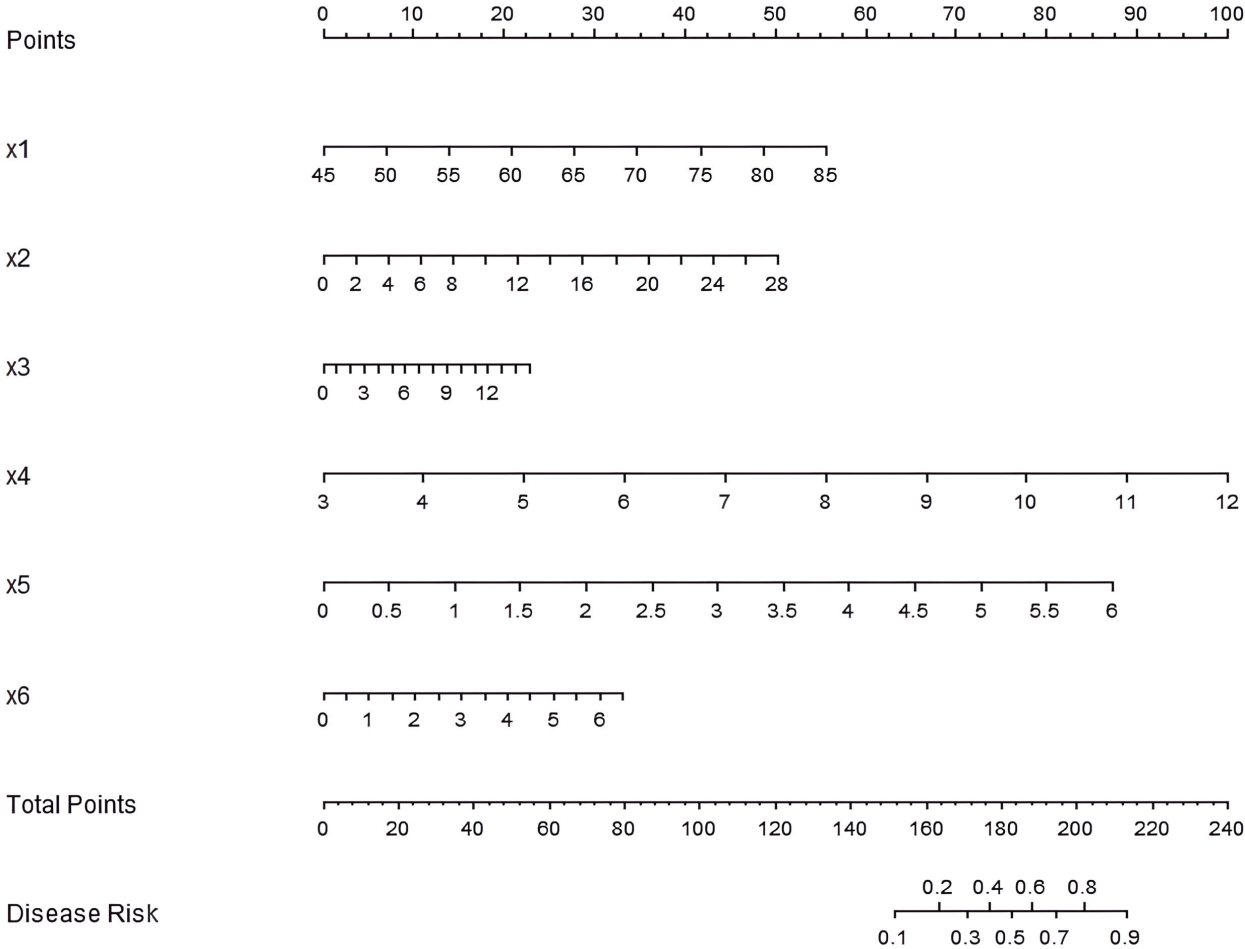
Nomogram of the prediction model for AD occurrence in diabetic patients. x1: age, x2: duration of diabetes, x3: HbA1c, x4: TG, x5: LDL-C, x6: HOMA-IR.

Based on the results of the multivariate Logistic regression analysis, a nomogram prediction model was constructed by including six independent risk factors: age, duration of diabetes, HbA1c, TG, LDL-C, and HOMA-IR (**Figure 1**). By assigning scores to each risk factor and calculating the total score, the risk probability of AD in diabetic patients was predicted. Specifically, a higher total score indicates a greater predicted risk of occurrence of AD.

To enhance the interpretability of the nomogram, a worked example is provided here. Consider a hypothetical diabetic patient with the following profile: Age = 65 years, Duration of diabetes = 12 years, HbA1c = 8.5%, TG = 2.2 mmol/L, LDL-C = 3.2 mmol/L, and HOMA-IR = 3.8. Using the nomogram in **Figure 1**: (1) For each variable, find the value on the corresponding axis and draw a line upward to the ‘Points’ axis to determine the individual score. (2) Sum all the points from the six variables to obtain the Total Points. (3) Locate the Total Points on the ‘Total Points’ axis and draw a line straight down to the ‘Risk of AD’ axis to read the predicted probability. In this example, the total points would be calculated, and the corresponding risk of AD might be, for instance, approximately 35%. This indicates that this patient falls into a high-risk category, warranting closer monitoring and proactive intervention. This step-by-step process allows clinicians to quickly estimate an individual patient’s risk and facilitate clinical decision-making.

### Validation and evaluation of the nomogram prediction model

**Figure 2:**
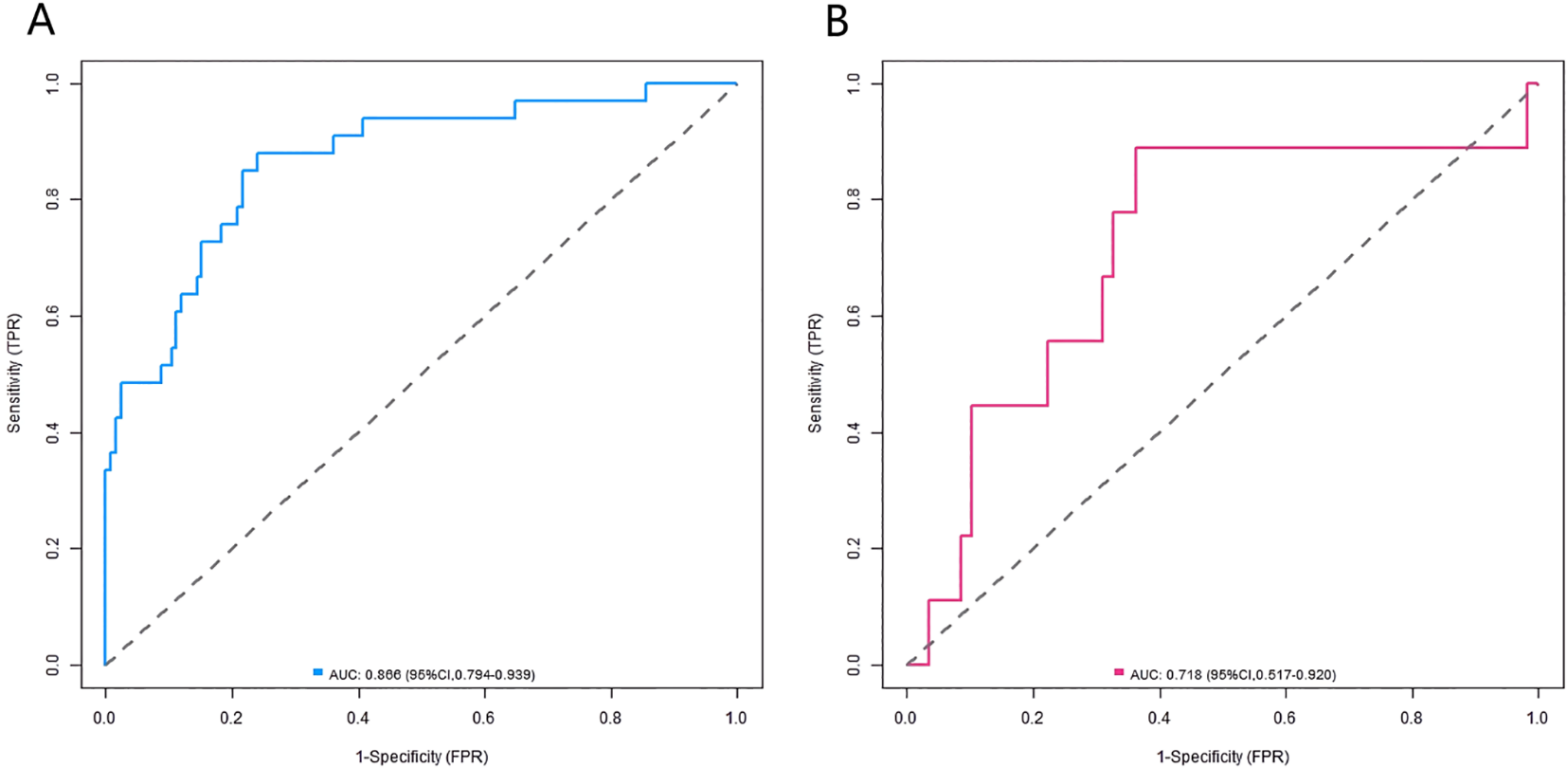
ROC curves in the training set **(A)** and the validation set **(B)**.

**Figure 3:**
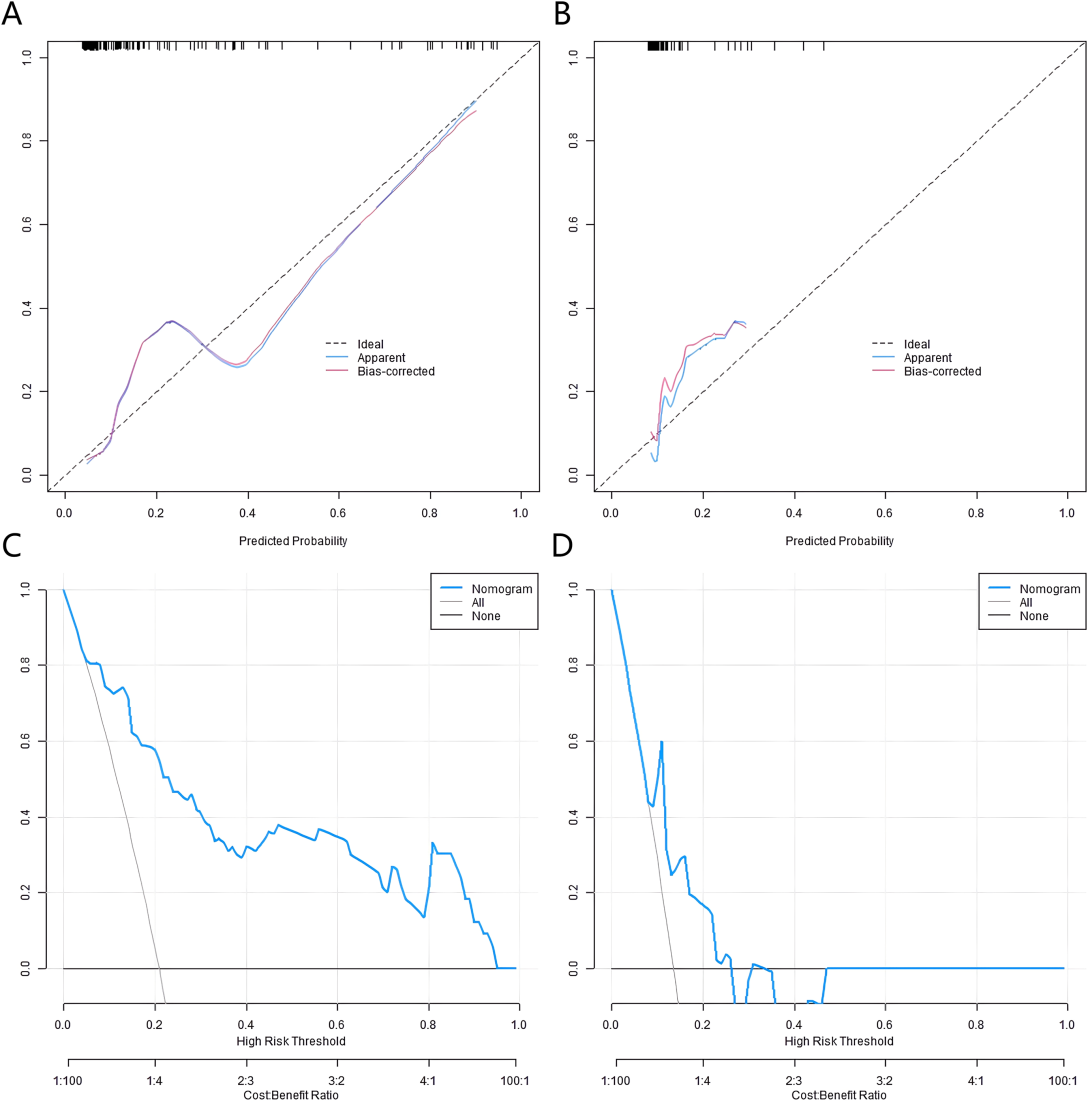
Calibration curves and decision curves in the training set **(A, C)** and the validation set **(B, D)**.

In the constructed training and validation datasets, the C-index of the developed nomogram prediction model reached 0.868 and 0.710, respectively. The ROC curves showed that the AUCs of the nomogram model for predicting AD occurrence in the training set and validation set were 0.866 (95% *CI*: 0.794-0.939) and 0.718 (95% CI: 0.517-0.920), respectively. The sensitivity and specificity were 0.879, 0.444 and 0.760, 0.862, respectively (**Figure 2**). The analysis results of the calibration curves showed that in the training set and validation set, the mean absolute errors between the model predicted values and the actual observed values were 0.103 and 0.116, respectively. The results of the Hosmer-Lemeshow test were *χ²* = 10.515, *P* = 0.230 and *χ²* = 5.987, *P* = 0.648, respectively (**Figures 3A, B**).

### Decision curve analysis

The results of the decision curve analysis revealed that when the Nomogram model was used to predict the risk of AD onset, and the set prediction probability threshold was in the range of 0.10 to 0.90, the net benefit rate of patients was greater than 0 (**Figures 3C, D**).

## Discussion

In this study, through multivariate regression analysis, age, diabetes duration, FPG, HbA1c, TG, and HOMA-IR were identified as independent risk factors for AD in diabetic patients. Age is a core risk factor for AD. Generally, the risk of developing AD increases with the growth of an individual’s age. As people age, various physiological functions of the body gradually decline and age, and the brain is no exception. The regenerative ability of nerve cells decreases, synaptic plasticity reduces, and at the same time, the function of the body’s antioxidant defense system weakens, and the resistance to various endogenous and exogenous damage factors decreases (7). In the context of diabetes, the cumulative damage effect of long-term hyperglycemia and disorder of glucose and lipid metabolism on the brain becomes more obvious with age, which further increases the risk of AD. The prolongation of diabetes duration means that patients are exposed to a hyperglycemic environment for a long time, and the damage of hyperglycemia to the neurovascular unit persists and gradually worsens (8). The integrity of the structure and function of the neurovascular unit is crucial for maintaining normal cognitive function. Long-term hyperglycemia can lead to nerve fiber degeneration and demyelination, damage to vascular endothelial cells, increased permeability of the blood-brain barrier, and then affect the exchange of substances in the brain and the transmission of nerve signals (9). These pathological processes will ultimately induce cognitive impairment (10). As important indicators reflecting blood glucose levels, the increase of FPG and HbA1c indicates poor blood glucose control. The persistent hyperglycemic state can promote the production and deposition of β-amyloid protein (Aβ), which forms senile plaques in the brain, one of the important pathological features of AD (11). At the same time, the hyperglycemic state can promote the abnormal hyperphosphorylation of tau protein, which in turn promotes the formation of neurofibrillary tangles, further destroying the structure and function of neurons. High TG can affect brain function through multiple pathways. On the one hand, it can increase blood viscosity, change hemodynamics, and reduce blood supply and oxygen supply to the brain. On the other hand, abnormal TG metabolism can trigger inflammatory responses and oxidative stress, damage nerve cells and glial cells, and affect neurotransmitter metabolism, ultimately leading to AD (12). Insulin resistance not only affects the glucose metabolism process but also interferes with insulin signaling in the central nervous system, thereby affecting the growth, survival, and function of nerve cells (13). Insulin resistance can trigger a series of subsequent metabolic dysfunctions, such as hyperglycemia and hyperlipidemia, which together increase the risk of AD.

The nomogram prediction model based on the clinical characteristics and glucose-lipid metabolism indicators of diabetic patients constructed in this study has important clinical significance and application value. The nomogram model integrates multiple independent risk factors, providing a highly intuitive and easy-to-operate assessment tool for clinicians to accurately evaluate the risk of AD in diabetic patients. By simply calculating and summing the scores corresponding to each risk factor, the patient’s risk score can be obtained, and then the probability of developing AD can be predicted (14). This helps to identify high-risk populations in the early stage in clinical practice, so as to implement individualized management for diabetic patients. For patients at high risk, clinicians can take more active intervention measures, such as strengthening blood glucose control, establishing stricter blood glucose management standards, improving and optimizing hypoglycemic treatment strategies, and selecting appropriate hypoglycemic drugs to reduce the damage of hyperglycemia to the brain. At the same time, actively regulating blood lipids, reducing the levels of TG, total cholesterol (TC), and low-density lipoprotein cholesterol (LDL-C), and increasing the level of high-density lipoprotein cholesterol (HDL-C) to improve the disorder of lipid metabolism can help reduce lipid peroxidation damage and inflammatory responses and protect nerve cells. For insulin resistance, measures such as improving lifestyle (e.g., reasonable diet, appropriate exercise) and using insulin sensitizers can be taken to enhance insulin sensitivity and optimize insulin signal transduction efficiency (15).

The evaluation of the nomogram model revealed a discernible decrease in discriminatory ability from the training set (C-index: 0.868, AUC: 0.866) to the validation set (C-index: 0.710, AUC: 0.718). This attenuation in performance, while not uncommon when moving from derivation to validation cohorts, underscores the preliminary nature of our model and highlights the imperative for further refinement before broad clinical deployment. Beyond the acknowledged limitation of sample size (16), this performance gap may also stem from the inherent biological complexity of the diabetes-AD nexus, which our current linear, main-effects model may not fully capture. To address this and guide future research, we propose several tangible steps for model enhancement. First, feature selection enhancement using techniques like LASSO regression or bootstrapping with variable selection could help create a more parsimonious and stable set of predictors. Second, given the plausible synergistic effects between risk factors (e.g., the effect of high HbA1c on AD risk might be amplified in the presence of high HOMA-IR), incorporating clinically relevant interaction terms should be explored. Third, the assumption of linear relationships between continuous predictors (like age or HbA1c) and log-odds of AD may be an oversimplification; employing restricted cubic splines or other methods to model potential non-linear relationships could significantly improve predictive accuracy. Finally, as previously noted, expanding the predictor set to include genetic, inflammatory, and cognitive reserve markers will be crucial to more comprehensively represent the multifactorial pathophysiology of AD in diabetics. According to the DCA, within the threshold probability range of 0.1-0.9, the net benefit of using the nomogram model for AD risk prediction in clinical decision-making was higher than the strategies of assuming that all patients would develop AD or none of them would. This result indicates that the model has practical value in clinical decision-making, providing valuable references for clinicians in formulating treatment strategies and intervention measures, helping them weigh the pros and cons of intervention measures, and formulating precise medical measures that can slow down the progression of the disease and improve the patients’ quality of life (17).

Although this study has achieved preliminary positive results, there are still some limitations. Firstly, this is a single-center, retrospective study with a modest sample size. Although our sample size met the statistical requirements for initial model development and we employed bootstrap internal validation to mitigate overfitting, the sample remains limited for a prediction model with six predictors. Therefore, external validation in a larger, multi-center cohort is essential to confirm the robustness and generalizability of our nomogram before it can be considered for clinical implementation (18). Secondly, this study primarily relied on routinely collected clinical and metabolic indicators. Consequently, several established risk factors for AD, such as apolipoprotein E (APOE) genotype, educational level (as an indicator of cognitive reserve), and specific systemic inflammatory markers, were not included in our model. The inclusion of these factors in future studies, leveraging larger multi-center cohorts with comprehensive data, is essential to build upon our initial model and develop a more precise and comprehensive predictive tool (19). Subsequent studies can further expand the scope of research factors, include more potential risk factors, and construct a more comprehensive and accurate prediction model. Third, a significant limitation of our study is the absence of a specific Mild Cognitive Impairment (MCI) cohort. MCI, particularly amnestic MCI, is often a prodromal stage of AD, and its identification is crucial for the earliest possible intervention (20). Diabetic patients have a markedly increased risk of developing MCI, with the pathophysiological continuum sharing profound overlaps with AD. Brain insulin resistance is a cornerstone of this link, leading to mitochondrial dysfunction, oxidative stress, and compromised synaptic plasticity, which manifest as MCI and progressively evolve into AD. Our model, which predicts the transition to full AD, likely captures the later stages of this cascade. Future studies should aim to develop prediction models that distinguish diabetic patients with normal cognition from those with MCI and those converting from MCI to AD. Incorporating biomarkers of insulin signaling and mitochondrial function, as suggested by this pathophysiological framework, could significantly enhance the predictive accuracy and clinical relevance of such models. Moreover, this study did not conduct long-term follow-up, so it was impossible to dynamically observe the patients’ condition changes and the long-term accuracy of the model prediction (21). In the future, longitudinal studies can be carried out to conduct regular follow-up on diabetic patients, evaluate the predictive performance of the model at different time points, and further explore the dynamic evolution relationship between diabetes and AD (22).

When considering the clinical implementation of this nomogram, it is important to acknowledge potential challenges, particularly regarding resource requirements. The model incorporates variables like HbA1c and HOMA-IR, which are standard in specialist endocrinology practice but may not be universally or readily available in all primary care settings, especially in resource-limited areas. The calculation of HOMA-IR, which requires fasting insulin measurement, can be a specific barrier due to its cost and limited availability. Furthermore, central insulin resistance disrupts mitochondrial function, leading to increased oxidative stress and bioenergetic deficits that compromise neuronal health and synaptic function, creating a vulnerable environment for cognitive decline (23). To enhance the model’s translational potential, future work could explore the development of a simplified version that utilizes more universally available measures (e.g., replacing HOMA-IR with fasting blood glucose alone or a clinical score for insulin resistance) without substantially compromising predictive accuracy. Alternatively, the current model could be positioned as a tool for use in secondary or tertiary care centers, where patients with diabetes are regularly followed up and these metabolic assessments are part of routine care. This pragmatic approach would facilitate the initial integration of risk stratification into clinical workflows, ensuring that the model reaches the patients who would benefit most from it.

In conclusion, this study constructed a nomogram prediction model for the occurrence of AD in diabetic patients, identified risk factors such as age. The model has good predictive performance and calibration and is of value in clinical decision-making. The results are helpful for early identification of high-risk populations, which is conducive to taking intervention measures to delay the progression of AD and improve the patients’ quality of life. However, due to the limitations of this study, it is necessary to carry out large-scale, multi-center prospective scientific studies, include more factors to improve the model, provide stronger support for prevention and treatment, and at the same time, deeply explore the pathogenesis and find new intervention targets.

## Data Availability

The raw data supporting the conclusions of this article will be made available by the authors, without undue reservation.
